# The nuclear localization sequence mediates hnRNPA1 amyloid fibril formation revealed by cryoEM structure

**DOI:** 10.1038/s41467-020-20227-8

**Published:** 2020-12-11

**Authors:** Yunpeng Sun, Kun Zhao, Wencheng Xia, Guoqin Feng, Jinge Gu, Yeyang Ma, Xinrui Gui, Xia Zhang, Yanshan Fang, Bo Sun, Renxiao Wang, Cong Liu, Dan Li

**Affiliations:** 1 Interdisciplinary Research Center on Biology and Chemistry, Shanghai Institute of Organic Chemistry, Chinese Academy of Sciences, Shanghai, 201210 China; 2 University of Chinese Academy of Sciences, Beijing, 100049 China; 3 State Key Laboratory of Bioorganic and Natural Products Chemistry, Center for Excellence in Molecular Synthesis, Shanghai Institute of Organic Chemistry, Chinese Academy of Sciences, Shanghai, 200032 China; 4 School of Life Science and Technology, ShanghaiTech University, Shanghai, 201210 China; 5 Department of Medicinal Chemistry, School of Pharmacy, Fudan University, Shanghai, 201203 China; 6 Bio-X-Renji Hospital Research Center, Renji Hospital, School of Medicine, Shanghai Jiao Tong University, Shanghai, 200240 China; 7 Bio-X Institutes, Key Laboratory for the Genetics of Developmental and Neuropsychiatric Disorders, Ministry of Education, Shanghai Jiao Tong University, Shanghai, 200030 China

**Keywords:** Biochemistry, Intrinsically disordered proteins, Cryoelectron microscopy

## Abstract

Human heterogeneous nuclear ribonucleoprotein A1 (hnRNPA1) serves as a key regulating protein in RNA metabolism. Malfunction of hnRNPA1 in nucleo-cytoplasmic transport or dynamic phase separation leads to abnormal amyloid aggregation and neurodegeneration. The low complexity (LC) domain of hnRNPA1 drives both dynamic phase separation and amyloid aggregation. Here, we use cryo-electron microscopy to determine the amyloid fibril structure formed by hnRNPA1 LC domain. Remarkably, the structure reveals that the nuclear localization sequence of hnRNPA1 (termed PY-NLS), which is initially known to mediate the nucleo-cytoplamic transport of hnRNPA1 through binding with karyopherin-β2 (Kapβ2), represents the major component of the fibril core. The residues that contribute to the binding of PY-NLS with Kapβ2 also exert key molecular interactions to stabilize the fibril structure. Notably, hnRNPA1 mutations found in familial amyotrophic lateral sclerosis (ALS) and multisystem proteinopathoy (MSP) are all involved in the fibril core and contribute to fibril stability. Our work illuminates structural understandings of the pathological amyloid aggregation of hnRNPA1 and the amyloid disaggregase activity of Kapβ2, and highlights the multiple roles of PY-NLS in hnRNPA1 homeostasis.

## Abstract (web summary)

Heterogeneous nuclear ribonucleoprotein A1 (hnRNPA1) shuttles between the nucleus and cytoplasm to regulate gene expression and RNA metabolism and its low complexity (LC) C-terminal domain facilitates liquid–liquid phase separation and amyloid aggregation. Here, the authors present the cryo-EM structure of amyloid fibrils formed by the hnRNPA1 LC domain, which reveals that the hnRNPA1 nuclear localization sequence forms the fibril core, and they discuss how ALS-causing mutations affect fibril stability.

## Introduction

Heterogeneous nuclear ribonucleoprotein A1 (hnRNPA1) is a multifunctional protein playing key roles in the regulation of gene expression and RNA metabolism^1–3^. It is involved in a wide range of RNA-processing events under normal and pathological cellular conditions including transcription, constitutive and alternative splicing, stability, nuclear export, and translation^4–7^. HnRNPA1 contains two globular RNA recognition motifs (RRM1 and RRM2) for the specific binding with mRNA precursor, followed by an unstructured low complexity (LC) C-terminal domain. HnRNPA1 LC consists of a cluster of RGG repeats with interspersed aromatic residues and a nuclear localization sequence (termed PY-NLS)^8–10^. HnRNPA1 LC is intrinsically disordered and exhibits a characteristic feature of homogenous and heterogeneous supermolecular polymerization^11–14^. It can mediate protein–protein and protein–RNA interactions to undergo liquid–liquid phase separation to participate in stress granule assembly^11,12^. The LC domain also exerts a prion-like function to mediate the amyloid aggregation of hnRNPA1, which is causative to neurodegenerative diseases including amyotrophic lateral sclerosis (ALS) and multisystem proteinopathy (MSP)^7,12,15–17^, and enhanced by inheritable mutations^12,15^.

HnRNPA1 predominantly localizes in the nucleus, while it shuttles between the nucleus and cytoplasm in response to cellular signals^11,18,19^. The transport of hnRNPA1 between the nucleus and cytoplasm is mediated by karyopherin-β2 (Kapβ2, also known as transportin-1) via direct binding of the PY-NLS sequence with Kapβ2^2,9,10^. The complex structure of Kapβ2 and PY-NLS reveals a high-affinity binding of PY-NLS in the C-terminal arch of Kapβ2^8^. Moreover, recent studies demonstrate that Kapβ2 engages PY-NLS or analogous NLSs to inhibit and even reverse the amyloid fibril formation of hnRNPA1 and other RNA-binding proteins^20^.

In this work, we determine the atomic structure of amyloid fibril formed by hnRNPA1 LC domain by cryo-electron microscopy (cryoEM). The structure reveals that the fibril core is mainly formed and stabilized by PY-NLS. Mutations of hnRNPA1 identified in familial ALS and MSP patients are all involved in the fibril core and contribute to fibril stability. This structure not only demonstrates the role of PY-NLS in driving hnRNPA1 amyloid aggregation at the atomic level but also renders mechanistic understanding for the anti-amyloid activity of Kapβ2.

## Results

### CryoEM structure determination of the amyloid fibril formed by hnRNPA1 LC domain

**Fig. 1:**
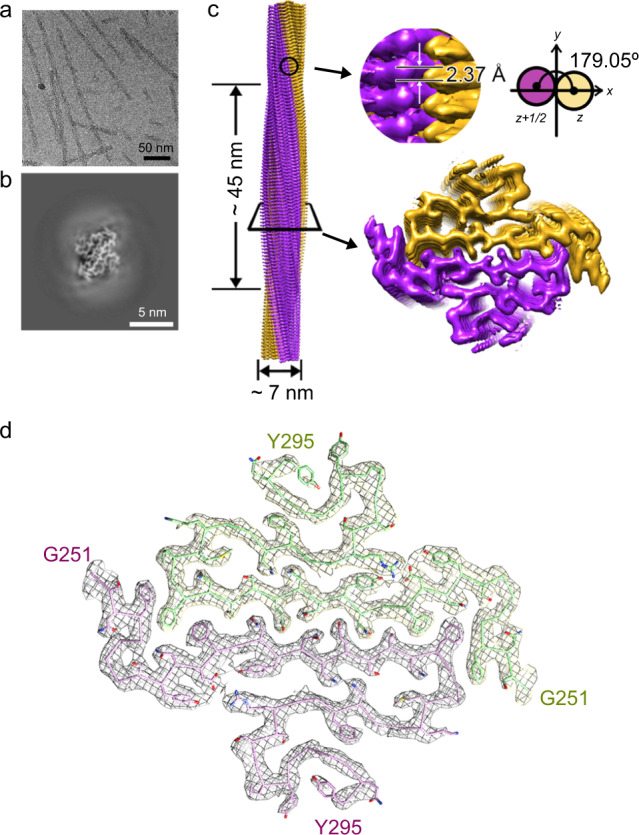
CryoEM structure determination of the amyloid fibril formed by hnRNPA1 LC domain. **a** CryoEM image of the amyloid fibrils formed by hnRNPA1 LC. Fibrils formed by hnRNPA1 LC purified in four independent experiments provide reproducible images. **b** CryoEM 3D reconstruction of the hnRNPA1 LC fibril. **c** CryoEM density map of the hnRNPA1 LC fibril. Fibril parameters including fibril width, half pitch, twist angle, and rise are marked. Protofilaments are colored in magenta and orange individually. The map was prepared by UCSF Chimera v1.13. **d** Cross-section view of the density map with structure model built upon it. Protofilaments are in different colors.

Recombinant hnRNPA1 LC (residues 186–320) was incubated at 16 °C for 2 days to form amyloid fibrils (Fig. 1a). The fibrils were irreversible as warmed up to 25 °C for 30 min (Supplementary Fig. 1a). Under this condition, the full-length hnRNPA1 also formed irreversible fibrils (Supplementary Fig. 1a). The prepared irreversible fibrils exhibited concentration-dependent toxicity to HEK293T cells (Supplementary Fig. 1b). The fibrils of hnRNPA1 LC were fixed on a carbon grid and frozen in liquid ethane. CryoEM data were collected by using a 300 keV Titan Krios microscope. Fibrils (62,656) from 3227 micrographs were selected for the reconstruction of hnRNPA1 LC fibril (Table 1). Two-dimensional (2D) classification showed one dominant species of left-handed twist fibril (~97% of the selected fibrils) (Supplementary Fig. 2). We then performed helical reconstruction by Relion^21^ and obtained the three-dimensioanl (3D) density map of the hnRNPA1 LC fibril to an overall resolution of 2.8 Å (Fig. 1b and Supplementary Fig. 3). The density map showed two protofilaments intertwining along an approximate twofold screw axis to form a left-handed helix with a width of ~7 nm and a half pitch of ~45 nm (Fig. 1c). The helical twist and rise between neighboring hnRNPA1 LC subunits are 179.05° and 2.37 Å, respectively (Fig. 1c and Table 1). Based on the high-resolution cryoEM density map, we were able to unambiguously build an atomic structure model for hnRNPA1 LC fibril (Fig. 1d). Despite that the fibrils were formed by hnRNPA1 residues 186–320, only residues 251–295 were involved in the fibril core (Fig. 1d).

**Table 1:** Statistics of cryoEM data collection and refinement.

Name	hnRNPA1 LC fibril
PDB ID	7BX7
EMDB ID	EMD-30235
Data collection	
Magnification	130,000
Pixel size (Å)	1.04
Defocus Range (μm)	−1.3 to −2.1
Voltage (kV)	300
Camera	K2 summit
Microscope	Titan Krios
Exposure time (s/frame)	0.25
Number of frames	32
Total dose (e^−^/Å^2^)	59
Reconstruction	
Micrographs	3227
Manually picked fibrils	62,656
Box size (pixel)	288
Inter-box distance (Å)	30.0
Segments extracted	801,707
Segments after Class2D	259,615
Segments after Class3D	69,037
Resolution (Å)	2.80
Map sharpening B-factor (Å^2^)	−109.473
Helical rise (Å)	2.37
Helical twist (°)	179.05
Atomic model	
Non-hydrogen atoms	1896
Protein residues	270
Ligands	0
r.m.s.d. Bond lengths	0.009
r.m.s.d. Bond angles	0.833
All-atom clash score	15.34
Rotamer outliers	0.00%
Ramachandran outliers	0.00%
Ramachandran allowed	9.30%
Ramachandran favored	90.70%

### Overall structural features of the hnRNPA1 LC fibril

**Fig. 2:**
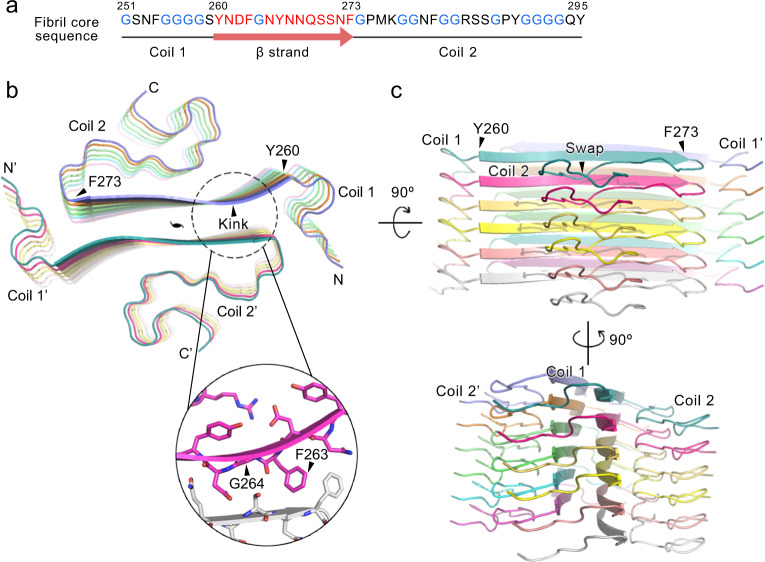
Overall structure of the hnRNPA1 LC fibril. **a** The primary sequence of the hnRNPA1 fibril core. Residues forming the β-strand are highlighted in red. Glycine residues are in blue. Secondary structures are indicated below. **b** Top view of the cryoEM structure of hnRNPA1 LC fibril. Six layers of backbones are shown. The structure is colored by chain. The pseudo-2-fold screw axis is indicated. The sheet kink region is zoomed in with side chains shown. **c** Side views of the fibril structure in **b**.

HnRNPA1 LC is rich in glycine residues dispersed throughout the sequence. This property is reflected in a high content of random coils in the fibril structure (Fig. 2a, b). Although the coil regions lack rigid secondary structures, they orderly stack along the fibril axis to enhance the stability of each protofilament (Fig. 2b, c). In particular, coil 2 at the C terminus swaps to the next subunit, to enhance the stability of protofilament (Fig. 2c); coils 1 and 2 form contacts between protofilaments, further enhancing the stability of the whole fibril (Fig. 2b, c).

The serpentine fold of hnRNPA1 fibril core remarkably contains a single but long β-strand composed of residues 260–273 (Fig. 2a, b). β-Strands of the same protofilament form a parallel β-sheet with a kink at _263_FG_264_ (Fig. 2b). A pair of β-sheets mate to provide major interactions between protofilaments for the fibril formation (Fig. 2b). Of note, ordered coil and kinked β-sheet structures have also been found in the reversible fibrils formed by FUS and hnRNPA1 segments^12,22–24^. Thus, these structural features might be general in Gly-rich LC domains.

### The hnRNPA1 LC fibril features a large interface between protofilaments

**Fig. 3:**
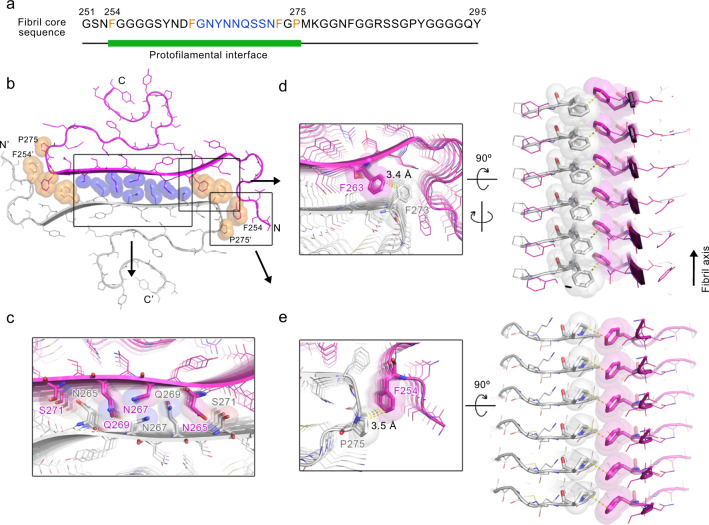
The interface between hnRNPA1 LC protofilaments. **a** The primary sequence of the hnRNPA1 fibril core. The green bar marks the region of the interface between protofilaments. Residues forming the steric zipper in the interface are highlighted in blue. Residues forming π-stacking and hydrophobic interactions flanking the steric zipper are highlighted in orange. **b** Top view of the hnRNPA1 LC fibril structure. Side chains that are involved in the interactions between protofilaments are shown in spheres. Among them, the polar side chains in the steric zipper are colored in blue; the Phe and Pro side chains are colored in orange. The two protofilaments are colored in magenta and gray, respectively. The framed regions are zoomed in and shown in top and side views in **c**–**e**.

Based on the fibril structure, residues 254–275, which represent half of the fibril core, are involved in the interface between protofilaments (Fig. 3a, b). The interface renders a buried surface area of 1814 Å^2^ per layer, which is larger than the reported fibril interfaces formed by various amyloid proteins such as α-synuclein and Tau (Supplementary Table 1). According to the local resolution estimation, the cryoEM density in this area is of the highest resolution of the entire fibril structure, indicating that the interface is the most stable region of the fibril (Supplementary Fig. 3b). The interface is tightly packed with a shape complementarity^25^ of 0.844 excluding any cofactor or water molecules. It is formed by a typical parallel in-register steric zipper in the middle sandwiched by Phe clusters (Fig. 3b). In the steric zipper, the opposing polar side chains of N265, N267, Q269, and S271 are tightly interdigitated to each other via van der Waals forces to form a shape complimentary dry interface (Fig. 3c), which is reminiscent of the first atomic structure of steric zipper formed by segment GNNQQNY of yeast prion Sup35^26^ (Supplementary Fig. 4). Flanking this steric zipper, F263 and the opposing F273 form T-shaped *π*-stacking and further stack along the fibril axis (Fig. 3a, b, d). Outside the *π*-stacking, F254 and the opposing P275 form hydrophobic interactions, which further enlarge the interface and, together with the *π*-stacking of F263-F273, stabilize the fibril by blocking the contact of solvent with the hydrophilic steric zipper interface (Fig. 3a, b, e).

### PY-NLS is the main component of the fibril core

**Fig. 4:**
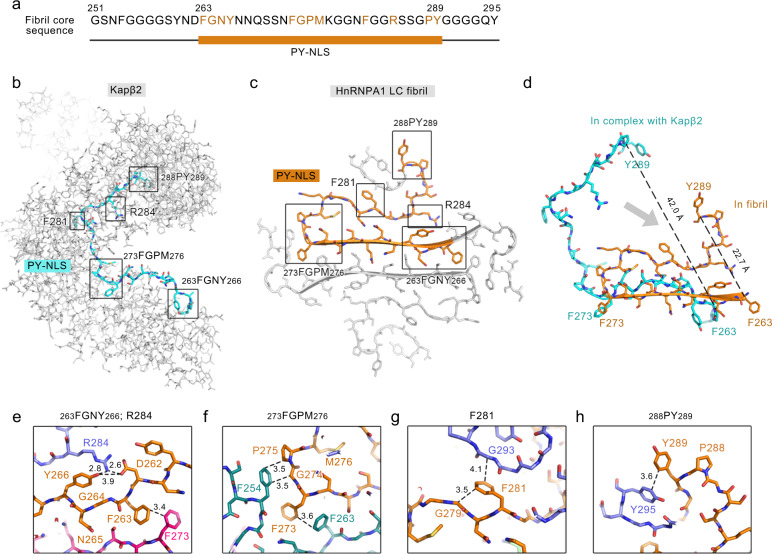
Structures of PY-NLS in complex with Kapβ2 and in the hnRNPA1 LC fibril. **a** The primary sequence of the hnRNPA1 fibril core. The orange bar marks the region of PY-NLS. Residues interacting with Kapβ2 are highlighted in orange. **b** Complex structure of PY-NLS and Kapβ2 (PDB ID: 2H4M). PY-NLS is shown in sticks and colored in cyan. Kapβ2 is shown in gray sticks. Regions of PY-NLS interacting with Kapβ2 are framed and labeled. **c** Top view of one layer of hnRNPA1 LC fibril structure. PY-NLS in one subunit is highlighted in orange. The rest of the structure is shown in gray sticks. The same framed regions of PY-NLS in **b** are framed and labeled. **d** Overlay of PY-NLS structures in complex with Kapβ2 and in fibril. **e**–**h** Zoom-in views of the framed regions in **c**. HnRNPA1 LC subunits are colored differently. Distances between side chains are indicated in Å.

PY-NLS is structurally disordered in free hnRNPA1. In the complex with Kapβ2, residues 263–289 of PY-NLS form an extended conformation to line in the C-terminal arch of Kapβ2^8^ (Fig. 4a, b). Strikingly, upon amyloid fibril formation, this same region forms the main part of fibril core (Fig. 4a, c). In contrast to the overall extended conformation in the complex with Kapβ2, PY-NLS forms a more compact structure in the fibril (Fig. 4d). F263-F273 further extends to a β-strand, referring to which the rest of PY-NLS curls into a serpentine fold (Fig. 4d). The distance between the backbones of the terminal residues reduces from 42.0 to 22.7 Å.

Moreover, PY-NLS residues that form extensive interactions with Kapβ2 also form important intra- and intermolecular interactions to stabilize the amyloid fibril structure. In detail, segments _263_FGNY_266_ and _273_FGPM_276_ that contact hydrophobic patches on Kapβ2 are essential for the formation of the interface between protofilaments (Fig. 4b, c, e, f). In addition to the *π*-stacking of F263 and F273 between protofilaments (Fig. 3d, e), and hydrophobic interaction between F254 and P275 (Fig. 3e, f) mentioned above, Y266 is involved in the intermolecular electrostatic interaction between D262 and R284, and forms *π*-cation interaction with R284 (Fig. 4e). This triad of D262, Y266, and R284 provides major forces to lock coil 2 with the β-strand (Supplementary Fig. 5). R284 also contributes to the binding of PY-NLS with Kapβ2 by forming salt links with the acidic interface of Kapβ2^8^ (Fig. 4b). Upon Kapβ2 binding, F281 and _288_PY_289_ also bind hydrophobic surfaces of Kapβ2. PY has been suggested as a consensus motif in various NLSs including hnRNP D, F, M, FUS, HuR, TAP, and PQBP-1, mutation of which validates the importance of this motif in the Kapβ2 binding^8,9,27–29^. Upon fibril formation, F281 forms intra- and intermolecular hydrophobic interactions with the main chains of G279 and G293 (Fig. 4g); F289 of the PY motif forms *π*-stacking with Y295 (Fig. 4h). These interactions provide important forces for the folding of coil 2 (Supplementary Fig. 5).

Therefore, these structures demonstrate that hnRNPA1 uses the same sequence of PY-NLS for Kapβ2 binding and amyloid aggregation. Thus, the binding of PY-NLS with Kapβ2 can directly prevent it from driving the amyloid fibril formation of hnRNPA1, which provides structural understanding for the chaperone activity of Kapβ2 in the inhibition and disaggregation of hnRNPA1 fibril formation^20^.

### Disease-causative hereditary mutations in the hnRNPA1 LC fibril structure

Several ALS- or MSP-causative familial mutations of hnRNPA1 have been identified including D262N/V, N267S, and P288S/A^15–17^. In terms of Kapβ2 binding for nucleo-cytoplasmic transport, the complex structure of PY-NLS and Kapβ2 shows the binding of residues 263–289 with Kapβ2^8^. Thus, being outside of the binding region, the D262N/V mutation is less likely to influence the cellular localization of hnRNPA1. In contrast, D262 forms salt bridge with R284 in the fibril structure (Fig. 4e and Supplementary Fig. 5). However, the role of this salt bridge in stabilizing the fibril structure is arguable. Previous structural study indicates that D262 is one of the key Asp residues that enable the reversibility of the hnRNAP1 fibril formed during liquid–liquid phase separation via charge repulsion^12^. In the presented irreversible fibril structure, the charge repulsion of aligned D262 along the fibril is neutralized by the aligned R284 (Fig. 4e and Supplementary Fig. 6). Upon D262N/V mutation, R284 may still form *π*-cation interaction with Y266 to maintain the same fibril structure (Fig. 4e). Indeed, we found that hnRNPA1 LC with D262N/V mutation formed amyloid fibrils with similar morphologies to that of the wild-type (WT) fibril in terms of fibril width and helical twist characterized by atomic force microscopy (AFM) and negative-staining transmission electron microscopy (TEM) (Supplementary Fig. 7a). On the other hand, the mutant fibrils were more stable than the WT calculated by Rosetta (Supplementary Table 2) and more resistant to proteinase K digestion (Supplementary Fig. 7b). These results are consistent with previous report that D262 mutations may remove the charge repulsion and enhance the formation of pathological irreversible fibril^12,30^. Collectively, the pathology of D262N/V mutation may mainly come from the impairment of reversible fibril formation during phase separation^12^, which consequently enhances pathological irreversible fibril formation.

N267 and P288 are involved in both Kapβ2 binding and fibril formation. Upon Kapβ2 binding, N267 forms intra- and intermolecular hydrophilic interactions with neighboring Q269 of hnRNPA1 and N803 of Kapβ2 (Supplementary Fig. 6b). Upon fibril formation, N267 locates at the center of the interface between protofilaments, and together with N265, Q269, and S271 forms steric zipper to stabilize the fibril interface (Fig. 3c and Supplementary Fig. 4). P288 is one of the key residues for Kapβ2 binding^8^ (Fig. 4b). In the fibril, although P288 exhibits no interaction with other residues, it is involved in the fibril core and stacks along the fibril (Supplementary Fig. 6a). Computational calculation by Rosetta indicated that mutations on both N267 and P288 sites destabilize the fibril structure (Supplementary Table 2). Thus, N267S and P288S/A mutations may influence both Kapβ2 binding and fibril formation.

## Discussion

**Fig. 5:**
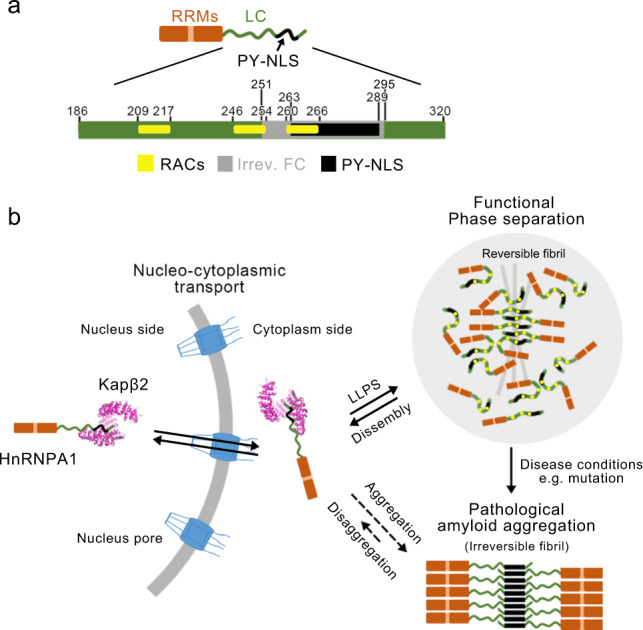
Hypothesis of hnRNPA1 forming different structures to adapt to different function. **a** Domain organization of hnRNPA1. The LC domain is colored in green and zoomed in. Segment 251–295 that is involved in the the irreversible fibril core (FC) is colored in gray. The PY-NLS (segment 263–289) is highlighted in black. The reversible amyloid-forming core segments (RACs) are highlighted in yellow. **b** Schematic illustration of the different structural states of hnRNPA1. HnRNPA1 monomer forms complex with Kapβ2 via PY-NLS. In the state of phase separation, the LC domain play a major role in mediating self-association of hnRNPA1 for liquid–liquid phase separation (LLPS) and reversible amyloid fibril formation. Under disease conditions, hnRNPA1 further assembles into irreversible amyloid fibrils via PY-NLS.

HnRNPA1 is one of the most abundant nuclear proteins and shuttles between nucleus and cytoplasm to regulate gene expression and RNA metabolism^2,4,5,31^. It is composed of 320 amino acids and nearly half of the sequence at the C terminus is of LC and intrinsically disordered. The LC domain underpins the nucleo-cytoplasmic transport, liquid–liquid phase separation, and amyloid aggregation of hnRNPA1^8,11,12,14,15^. Our work reveals that the PY-NLS in the LC domain of hnRNPA1 is key not only for the nucleo-cytoplasmic transport but also for the self-association of amyloid fibril formation. Intriguingly, hnRNPA1 can form both reversible and irreversible amyloid fibrils (Supplementary Fig. 1a). The former is suggested to play a functional role in the phase separation of hnRNPA1^12^, whereas the latter is pathologically associated with neurodegeneration^11,15^. Our previous work has identified three reversible amyloid-forming core segments (so-called RACs) of hnRNPA1, which share consensus motifs of (N)D(N) and (G)F/Y(G), and form highly reversible amyloid fibrils^12^ (Supplementary Fig. 8a). We have also shown that truncation of RAC3, but not RAC1 and RAC2, completely eliminates the irreversible amyloid fibril formation of hnRNPA1^12^. Consistent with this result, the current work showed that RAC3 resides in the irreversible fibril core formed by the hnRNPA1 LC domain (Fig. 5a and Supplementary Fig. 8a). Moreover, under the condition that the LC domain formed irreversible fibrils, full-length hnRNPA1 also formed irreversible fibrils and both fibrils exhibited cytotoxicity in a concentration-dependent manner (Supplementary Fig. 1). In addition, the two fibrils exhibited similar degradation patterns at low molecular weights as treated with proteinase K (Supplementary Fig. 8b). Collectively, these data indicate that the irreversible fibril structure formed by the LC domain presented in this work may represent that formed by the full-length protein.

Recently, Eisenberg and colleagues^32^ reported a reversible fibril structure formed by hnRNPA2 LC. The sequence of hnRNPA1 LC and hnRNPA2 LC are similar, and their fibrils appear to involve a similar region to form fibril core (Supplementary Fig. 9a). However, the reversible fibril of hnRNPA2 LC represents a single protofilament, which lacks the large interface between protofilaments as seen in the irreversible fibril of hnRNPA1 LC (Supplementary Fig. 9b). Thus, the two fibril structures together may shed light on the structural transition from reversible to irreversible fibrils.

Combining previous and the current work, we hypothesize that hnRNPA1 forms different structures via its LC domain to adapt to different functions. Under physiological conditions, hnRNPA1 shuttles between the nucleus and cytoplasm via its PY-NLS forming an extended structure lining in Kapβ2 (Fig. 5b). In response to cellular stimuli, the LC domain self-associates via multivalent interactions^11,14^ and reversible amyloid interactions^12,14,30^, to drive phase separation and incorporation of hnRNPA1 into stress granules (Fig. 5b). Under pathological conditions such as hereditary mutations, decreased binding affinity with Kapβ2 or disruption of phase separation dynamics may expose hnRNPA1 LC, in particular the PY-NLS, and thus contribute to the pathological amyloid fibril formation and neurodegeneration (Fig. 5b).

## Methods

### Protein expression and purification

The genes encoding hnRNPA1 LC (residues 186–320) or mutants were inserted into pET32M vector with a Trx1 tag. HnRNPA1 LC was overexpressed in *Escherichia coli* BL21 (DE3) at 25 °C overnight after induction by 0.4 mM isopropyl-1-thio-d-galactopyranoside (IPTG). Cells were collected by centrifugation and lysed in the lysis buffer (50 mM Tris-HCl and 6 M guanidine hydrochloride, pH 8.0) at room temperature. Cell lysate was sonicated for 30 min, followed by centrifugation at 30,966 × *g* for 45 min at 4 °C. The supernatant was loaded into Ni column (HisTrap FF, GE Healthcare) and HnRNPA1 LC was eluted with the elution buffer containing 50 mM Tris-HCl, 6 M guanidine hydrochloride, and 50 mM imidazole pH 8.0. The protein was further purified by high-performance liquid chromatography (HPLC) (Agilent 1260 Infinity, Agilent Technologies), with the elution buffer containing 20–80% (v/v) acetonitrile. After freeze-drying, the purified hnRNPA1 LC was resuspended with 50 mM Tris-HCl, 100 mM NaCl pH 7.5. The Trx1 tag was cleaved by 3C enzyme with a 50 : 1 (protein : enzyme) molar ratio at room temperature overnight. After centrifugation at 3724 × *g* for 30 min at room temperature, the precipitate was dissolved by the lysis buffer. The hnRNPA1 LC protein without tag was obtained by additional purification by HPLC and freeze-dried.

Full-length hnRNPA1 was expressed in *E. coli* BL21 (DE3) pLysS at 25 °C overnight after induction with 0.4 mM IPTG. Cells were lysed followed by centrifugation at 30,966 × *g* for 45 min at 4 °C. The supernatant was subjected to SP FF column (GE Healthcare) in 50 mM Tris-HCl, 100 μg/ml RNase A, 2 mM dithiothreitol (DTT), 1 mM phenylmethylsulfonyl fluoride (PMSF) pH 7.5. The bound protein was eluted with 50 mM Tris-HCl, 300 mM NaCl, 2 mM DTT pH 7.5 and, after concentration, was subjected to Superdex 75 16/60 (GE Healthcare) in 50 mM Tris-HCl pH 7.5, 500 mM NaCl, and 2 mM DTT.

All primers used in this work are listed in Supplementary Table 3.

### Fibril formation

hnRNPA1 LC (25 μM) in the buffer containing 50 mM Tris-HCl, 100 mM NaCl pH 7.5 was incubated at 16 °C for 2 days without agitation. The fibrils were collected and dialyzed into 10 mM acetate buffer (pH 5.0) overnight for cryoEM sample preparation.

### Negative-staining TEM

Four microliters of fibril sample were incubated on a glow-discharged copper grid (200 mesh, Beijing Zhongjingkeyi Technology Co., Ltd) for 45 s and then washed by an aliquot of deionized water and 3% w/v uranyl acetate, followed by staining with an aliquot of 3% w/v uranyl acetate for another 45 s and dried in air. Images were captured by Tecnai T12 microscope (FEI Company, 120 kV).

### Lactic acid dehydrogenase assay

HEK293T cells were treated with hnRNPA1 LC, full-length hnRNPA1, and α-synuclein preformed fibrils at indicated concentrations, respectively. The cells were collected 2 days after treatment. The lactic acid dehydrogenase assay was performed following the manufacturer’s instructions. The absorbance was recorded at 490 and 680 nm (background) with BioTek Synergy 2 Multi-Mode Microplate Readers (Thermo Scientific). Statistical significance was calculated by one-way analysis of variance using GraphPad Prism.

### CryoEM data collection

HnRNPA1 LC fibrils were loaded onto glow-discharged copper Quantifoil grids (R1.2/1.3, 300 mesh) and then plunge-frozen in liquid ethane precooled by liquid nitrogen using Vitrobot Mark IV (FEI). Micrographs were captured using a Gatan K2 Summit camera on a Titan Krios transmission electron microscope (FEI) operated at 300 kV with a GIF Quantum energy filter (slit width 20 eV) and collected in dose fractionation mode (32 frames per micrograph) by Serial EM software^33^. A magnification of ×130,000 with physical pixel size 0.52 Å in super-resolution mode was applied. More details are shown in Table 1.

### Imaging processing

Motion correction implement of MotionCorr2 was used to correct beam-induced motion of movie frames with dose-weighting implement^34^. All 32 frames per micrograph were aligned with 1.356 Å physical pixel size. CTFFIND4.1.8 was then used to estimate the contrast transfer function^35^. All filaments were picked manually using manual picking method of RELION3.0^21^.

### Helical reconstruction

Helical reconstruction was performed by RELION3.0^21^. A box size of 288 pixels with an inter-box distance of 30 Å was used in extraction method and 801,707 segments were obtained for subsequent reference-free 2D classification. A staggered arrangement was observed in 2D classification results, suggesting a pseudo-2_1_ symmetry. An initial helical rise of 2.4 Å and helical twist of 179.01° calculated by the apparent crossover distance of filaments were set in 3D classification. Segments (259,615) selected after 2D classification were used to construct an initial 3D model de novo. The initial 3D model low-pass filtered to 60 Å was then applied as reference map in 3D classification. Local search of symmetry to optimize of helical twist and rise was not carried out until separation of β-strands along the helical axis was shown in 3D classification. Several rounds of 3D classification with *K* = 3 was performed to remove heterogeneous segments. Optimized parameters and selected segments were applied for 3D refinement and final reconstruction with appropriate reference. Post-processing with a soft-edge solvent mask in 20% central Z length was performed to sharpen final reconstruction. Fourier shell correlations at 0.143 criterion were used to estimate the resolution of the final map. A soft spherical mask without masking a 3D helix was used to gain local resolution estimates.

### Atomic model building and refinement

The central region of the density map, which exhibits the highest resolution, was used to build atomic model de novo in COOT^36^. The model containing three layers was generated and then refined by PHENIX using the real-space refinement program^37^.

### Buried surface area calculation

The buried surface area was calculated using areaimol from the CCP4 package^38^. with a probe radius of 1.4 Å. The difference between the solvent accessible surface areas of one hnRNPA1 LC chain alone and within the five-layer fibril structure represents half of the reported buried area.

### Atomic force microscopy

Four microliters of fibril sample were loaded onto a clean mica surface for 3 min at room temperature and washed by deionized water to remove unbound fibrils. The sample was then dried in air and probed in air on scanAsyst mode using Multimode 8 scanning probe microscope (Bruker). SCANASYST-Air probe with a spring constant of 0.4 N/m and a resonance frequency of 70 kHz (Bruker) was used to carry measurements. Images were captured at a fixed resolution (512 × 512 data points) at a 1 Hz scan rate. Supplied software (NanoScope Analysis 1.5, Bruker) was used to process images and height.

### Calculation of fibril stability

Two modules, ddg_monomer^39^ and Cartesian_ddG^40^ of Rosetta software, were used to calculate the stability of the fibrils. For ddg_monomer, we first performed energy minimization on the prepared fibril structure to generate a distance restraints file. We used high-resolution protocols to run the ddg_monomer program and Rosetta standard side chain optimization module Packer to optimize the rotamers of all residues. Then, three rounds of gradient-based minimization were performed, in which the van der Waals repulsion term weights were sequentially reduced. A total of 50 structural optimizations were performed on the WT and mutant fibril structures. The top three optimal structures were used to calculate the ΔΔG. For Cartesian_ddG, the fibril structure was first relaxed in cartesian space with backbone and side chain restrained, and the program was run in protein stability mode. Three amino acid backbone degrees of freedom adjacent to the mutated residue were considered in the calculation. The van der Waals and solvation energy terms have a cutoff value of 9 Å. The difference in averaged over five rounds for WT and mutant fibril was taken as ΔΔG.

### Proteinase K digestion

hnRNPA1 LC WT and mutant fibrils (2.5 μg) were subjected to digestion. Before digestion, the fibrils in phosphate-buffered saline (PBS) buffer were sonicated at 20% power for 15 times (1 s per time, 1 s interval) on ice by JY92-IIN sonicator (Xinyi Sonication Equipment Company, Ningbo, China). For proteinase K digestion, the fibrils were incubated with proteinase K (Invitrogen) in PBS buffer at 37 °C for indicated time. The reaction was terminated by adding 1 mM PMSF. After digestion, the samples were boiled with SDS-loading buffer for 15 min and then loaded on the 4–20% Bis-Tris gel (GenScript). The gel was stained with Coomassie brilliant blue and imaged with Image Lab 3.0 (Bio-Rad).

### Reporting summary

Further information on research design is available in the Nature Research Reporting Summary linked to this article.

## Supplementary information

Supplementary Information

Reporting Summary

## Data Availability

Density maps of the hnRNPA1 LC fibril are available through EMDB with entry code: EMD-3025. The structural model was deposited in the Protein Data Bank with entry code: 7BX7. Other structural models used in this study are available in the Protein Data Bank with entry codes as follows: 2H4M (X-ray diffraction structure of Kapβ2), 1YJP (X-ray diffraction structure of GNNQQNY from yeast prion Sup35), 6RT0 (α-synuclein polymorph 1a), 6CU8 (α-synuclein polymorph 1b), 6XYO (α-synuclein MSA type I), 6XYP (α-synuclein MSA type II), 6TJX (Tau CBD type II), 6NWP (Tau CTE type I), and 6NWQ (Tau CTE type II). Source data are provided with this paper. Other data that support the findings of this study are available from the corresponding authors upon reasonable request.

## Source data

Source Data
