# Supplementary material for: The nuclear localization sequence mediates hnRNPA1 amyloid fibril formation revealed by cryoEM structure
Source: Nat Commun. 2020 Dec 11;11:6349. doi: 10.1038/s41467-020-20227-8 (PMC7733464; doi:10.1038/s41467-020-20227-8)
Supplement: Supplementary file 1 — Supplementary Information [file 41467_2020_20227_MOESM1_ESM.pdf]

Supplementary Information

The nuclear localization sequence mediates hnRNPA1 amyloid fibril formation revealed by cryoEM structure

Yunpeng Sun, Kun Zhao et al.

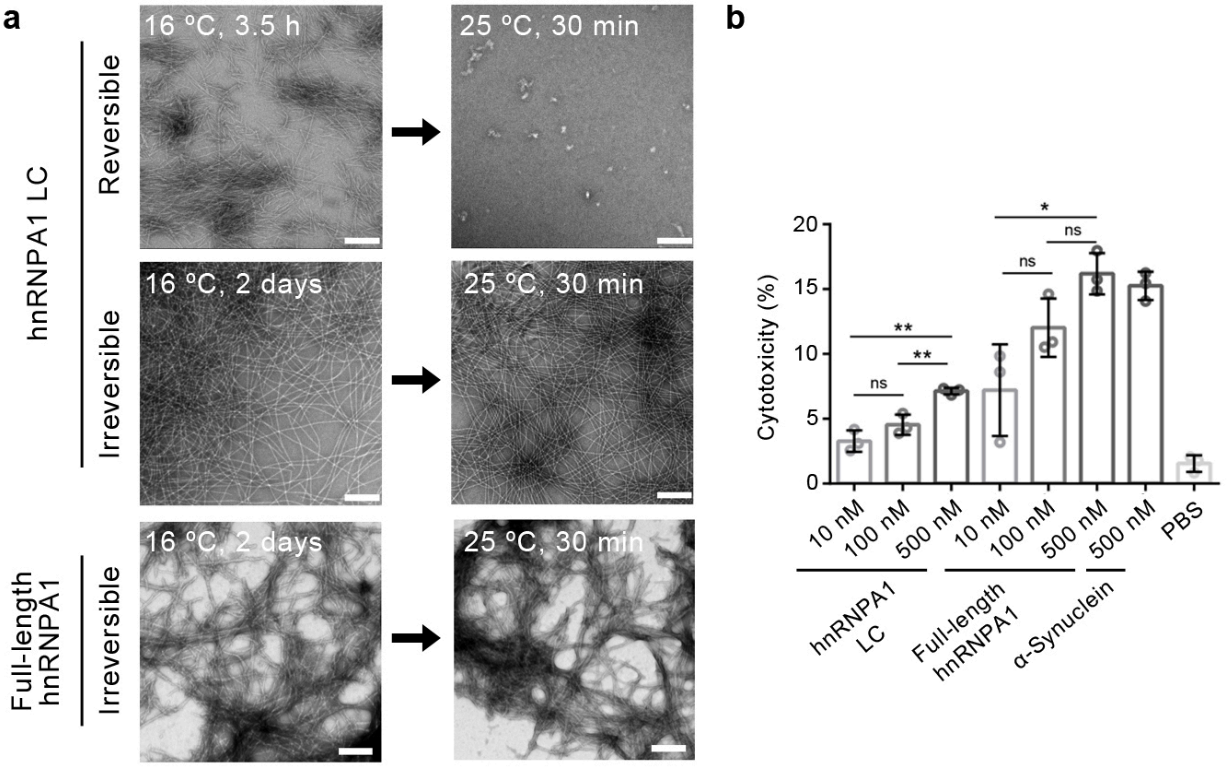

Supplementary Figure 1 Fibril formation and cytotoxicity. a, TEM images of amyloid fibrils formed by hnRNPA1 LC and full-length hnRNPA1. Scale bar: 300 nm. The irreversible fibrils of hnRNPA1 LC were used for structure determination in this work. The images represent reproducible results in >3 independent experiments. b, Cytotoxicity of the irreversible fibrils of hnRNPA1 LC and full-length hnRNPA1 to HEK 293T cells by the LDH assay. α-Synuclein serves as positive control. PBS serves as blank control. Data are shown as mean ± S.D., with n = 3 biologically independent samples. *p <0.05 (0.0128); **p < 0.01 (hnRNPA1 LC 10 nM vs. 500 nM: 0.0010; 100 nM vs. 500 nM: 0.0079); ns, not significant. One-way ANOVA followed by Tukey HSD post hoc test.

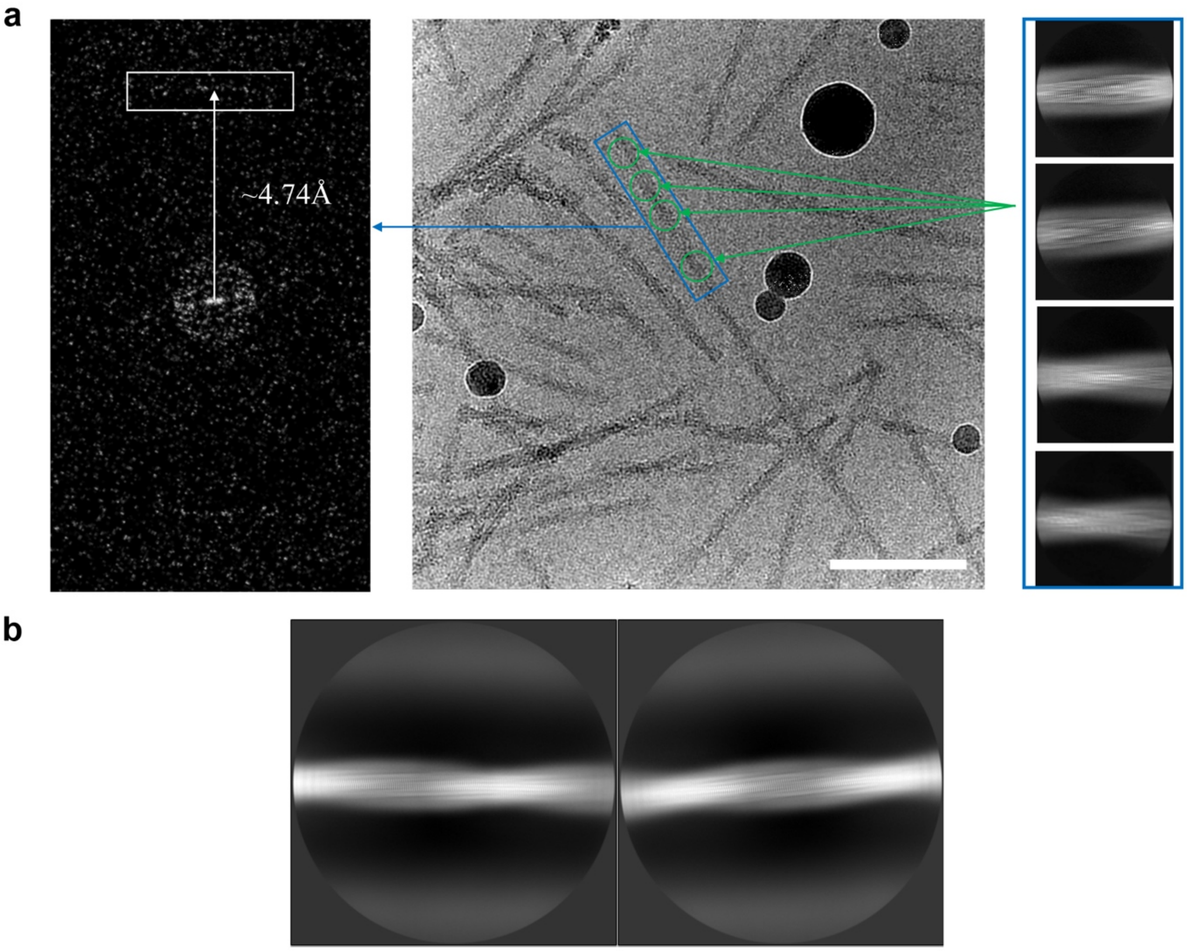

Supplementary Figure 2 2D classification of the hnRNPA1 LC fibril. a, The power spectra of indicated fibrils on the left are generated by ImageJ 2.0.0. The layer line (pointed by arrow) corresponds to the helical rise. CryoEM micrographs are shown in the middle. Scale bar = 100 nm. Fibrils formed by hnRNPA1 LC purified in 4 independent experiments provide reproducible images. 2D class averages are shown on the right. Box size: 288 pixels. b, 2D class averages show left-handed twist of the fibril. Box size: 686 pixels.

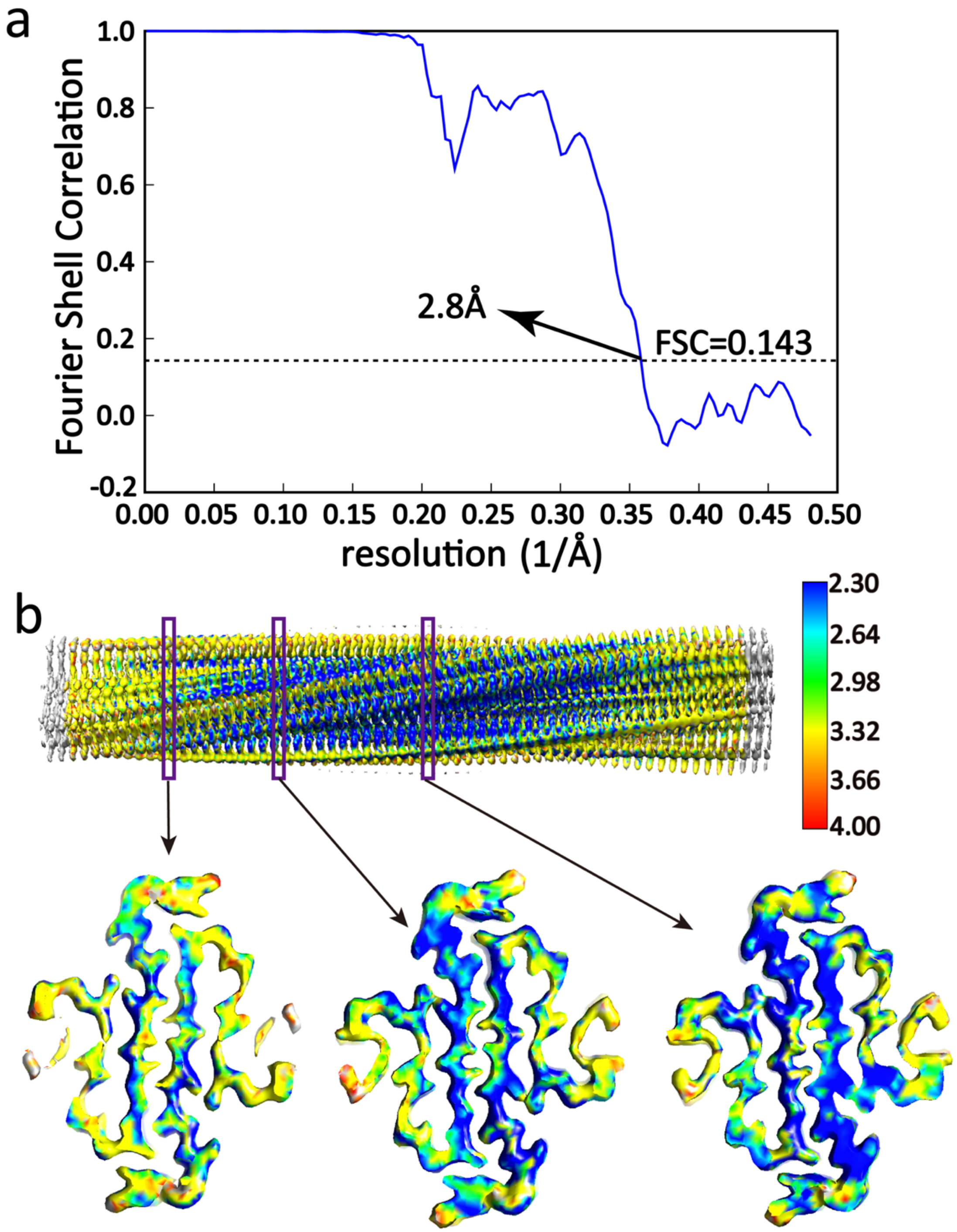

Supplementary Figure 3 Resolution estimation of the cryoEM structure of hnRNPA1 LC fibril. a, Gold-standard Fourier shell correlation (FSC) curve of the hnRNPA1 LC fibril. The overall resolution is 2.8 Å. b, Local resolution estimation. EM reconstruction maps are colored based on the local resolutions. Color scale indicating the resolution range is shown. Cross sections of the fibril at three interspersed positions are shown.

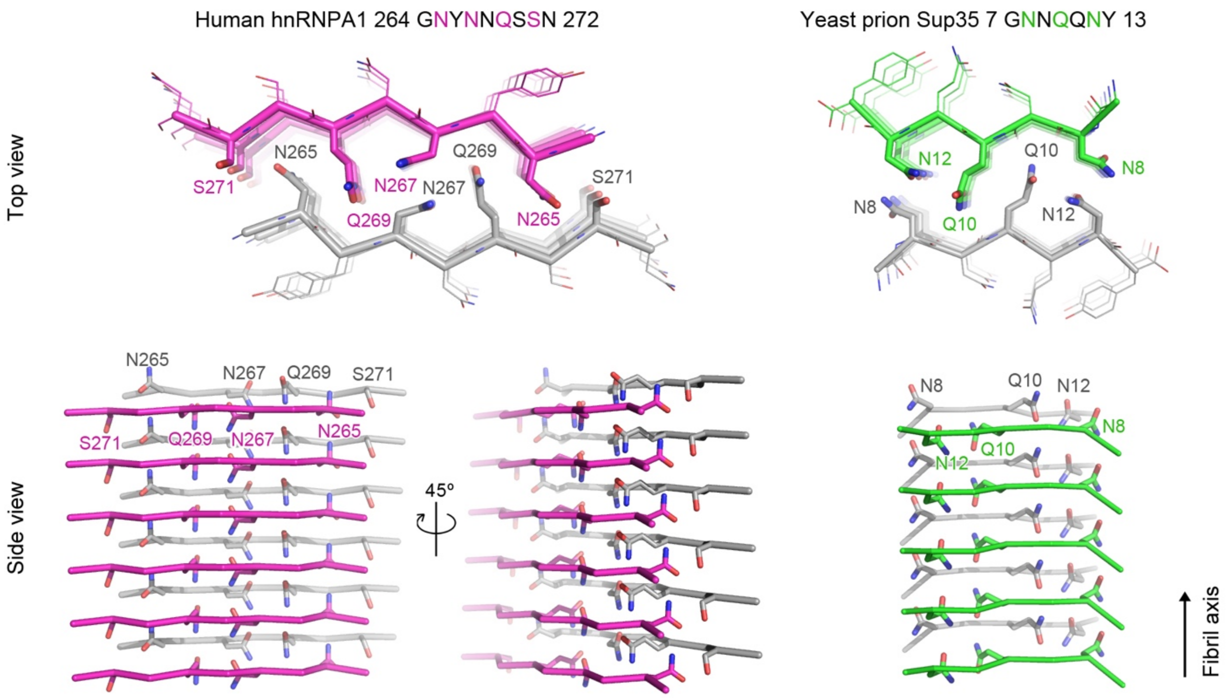

Supplementary Figure 4 Steric zippers formed by human hnRNPA1 and yeast prion Sup35 fragments. The structure of hnRNPA1 segment is extracted from the fibril structure of hnRNPA1 LC. The structure of Sup35 segment is the crystal structure of peptide GNNQQNY (PDB ID: 1YJP). The structures are colored differently by β sheet. Residues that are involved in inter-sheet interactions are highlighted in the sequences. Their side chains are labeled and shown in sticks in the structures; others are shown in lines.

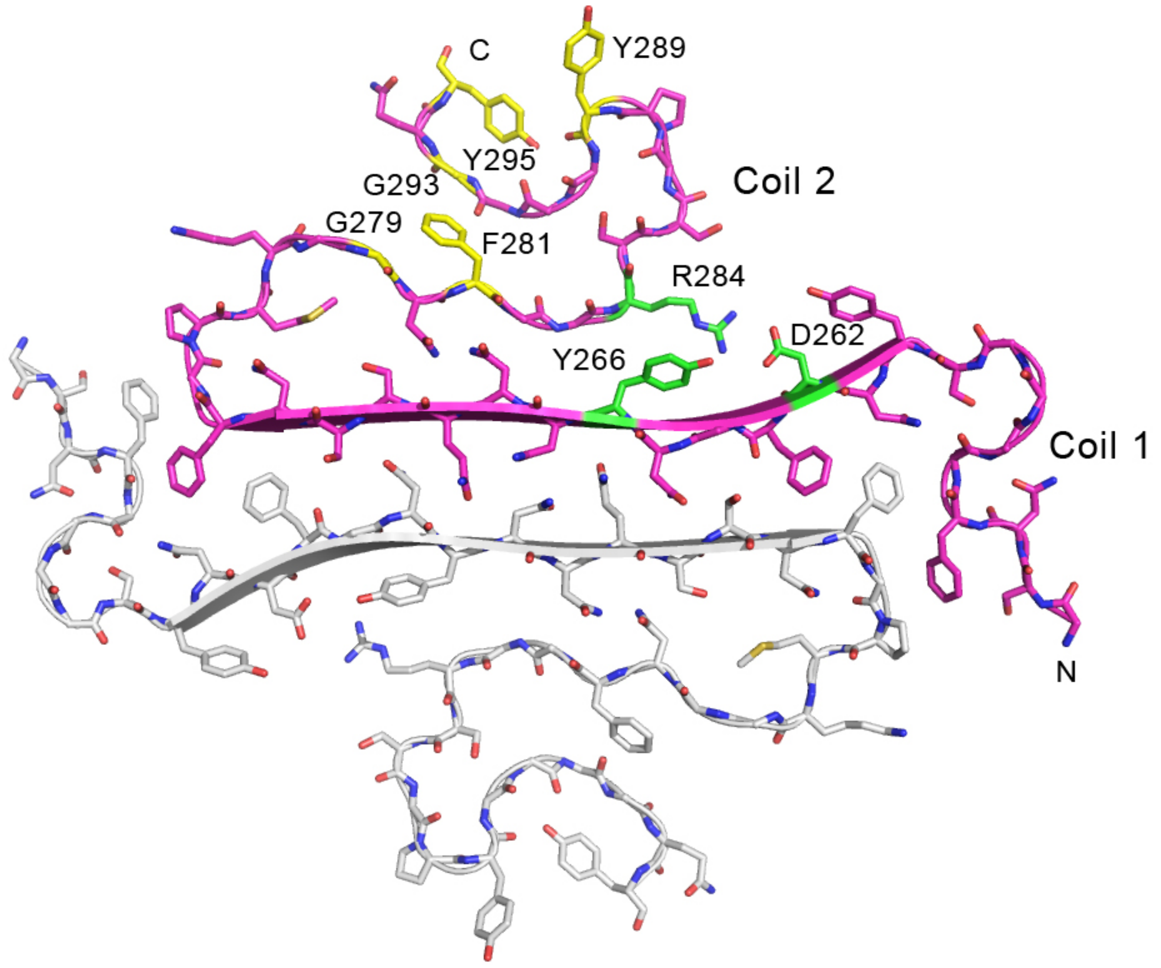

Supplementary Figure 5 Major interactions for coil 2 stabilization. One layer of the hnRNPA1 LC fibril structure is shown. The structure is shown in sticks and cartoon. The two hnRNPA1 LC subunits are colored in magenta and gray, respectively. Triad residues of D262-Y266-R284 are highlighted in green. Interacting residues G279-F281-G293 and Y289-Y295 are highlighted in yellow.

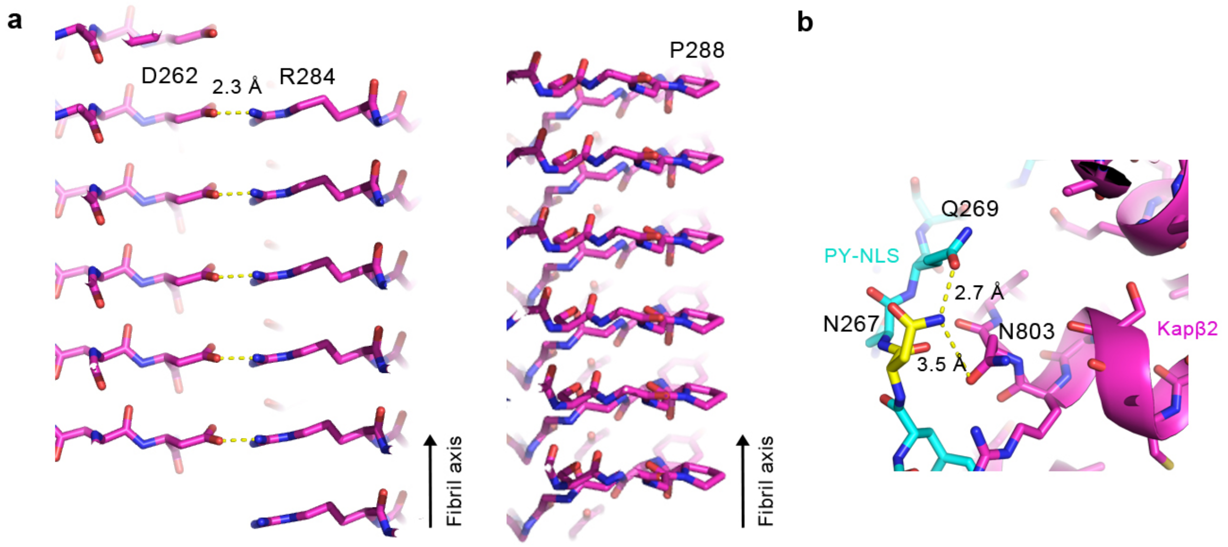

Supplementary Figure 6 Snapshots of the structures of hnRNPA1 LC fibril and Kapβ2 and PY-NLS complex. a, Side views of the electrostatic interactions between D262 and R284 residues (left), and stacking of P288 residues along the fibril axis (right). b, Intra-molecular Interactions of PY-NLS between N267 and Q269, and inter-molecular interactions between N267 of PY-NLS and N803 of Kapβ2.

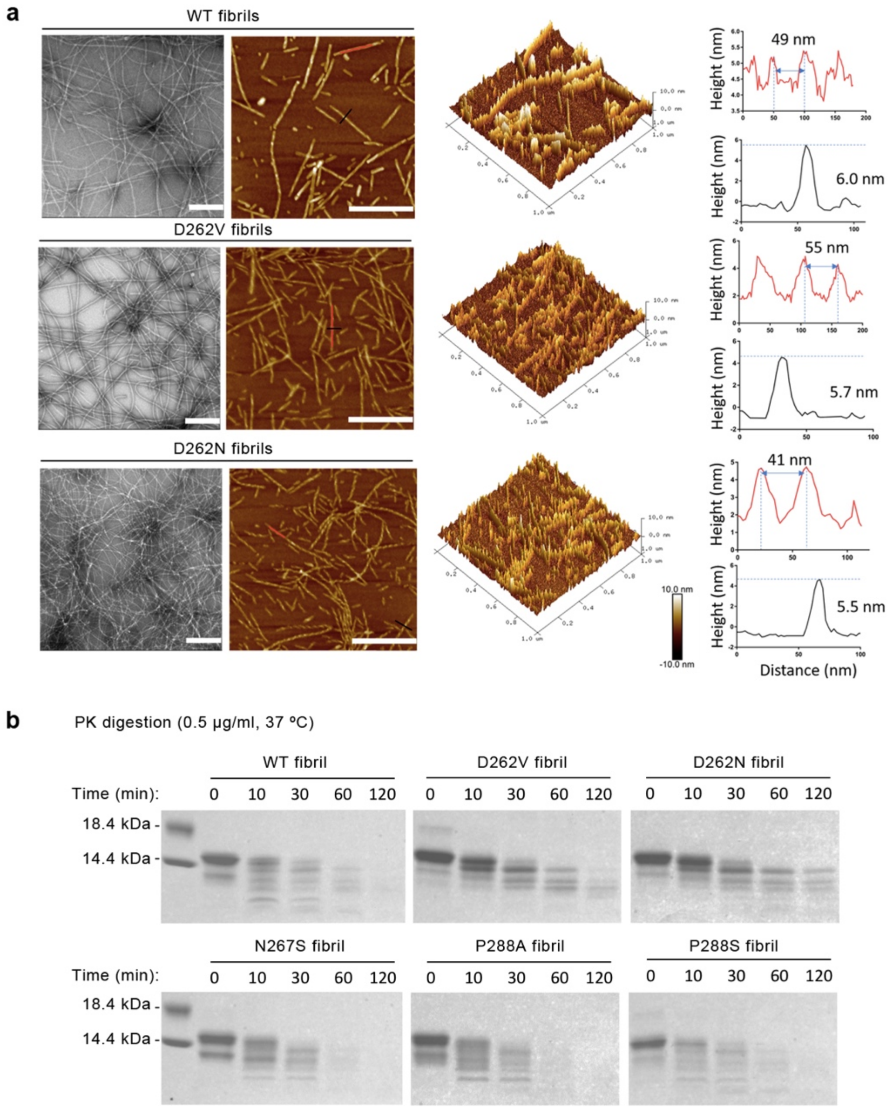

Supplementary Figure 7 Comparison of the fibrils formed by hnRNPA1 LC and variants. a, Characterization of fibril morphology by negative-staining TEM (left gray images) and AFM (right brown images). Scale bar: 300 nm. 2D and 3D AFM images are shown. Analyses of the cross section and along the fibril based on the AFM images are indicated with black and red lines, respectively. Fibrils formed by different proteins purified in 3 independent experiments provide reproducible images. b, SDS-PAGE gels of the hnRNPA1 WT and mutant fibrils digested by proteinase K (PK). The fibrils were sonicated before digestion. The result is reproducible in 3 independent experiments.

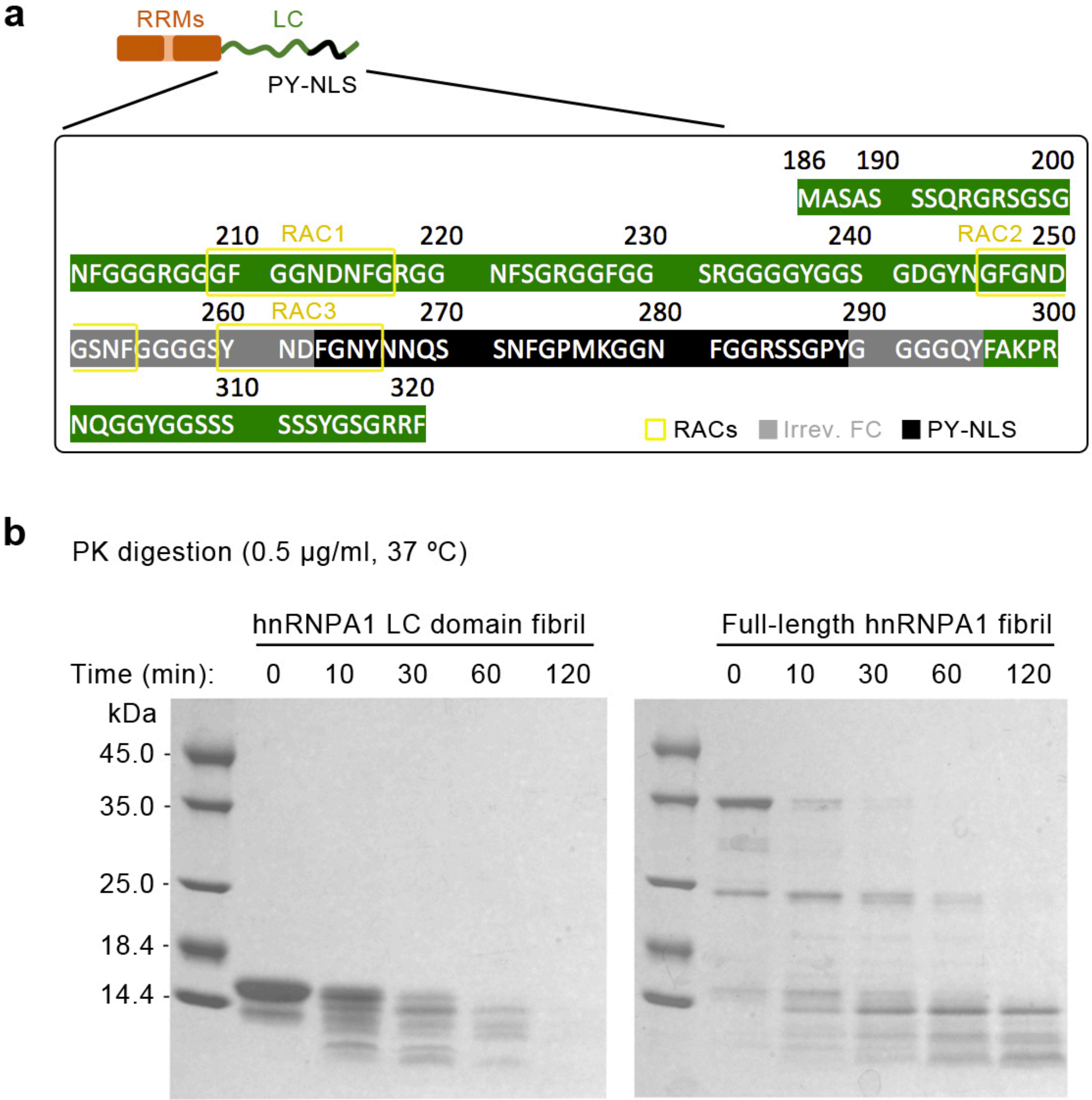

Supplementary Figure 8 RAC3 is essential for the irreversible fibril formation of both full-length and the LC domain of hnRNPA1. a, Domain organization of hnRNPA1. The sequence of LC domain is shown and highlighted in green. The reversible amyloid-forming core segments (RACs) are framed with yellow boxes. The segment that is involved in the the irreversible fibril core (FC) of hnRNPA1 LC is shaded in gray. The PY-NLS is shaded in black. b, SDS-PAGE gels of the full-length and LC domain of hnRNPA1 fibrils digested by proteinase K (PK). The fibrils were sonicated before digestion. The result is reproducible in 3 independent experiments.

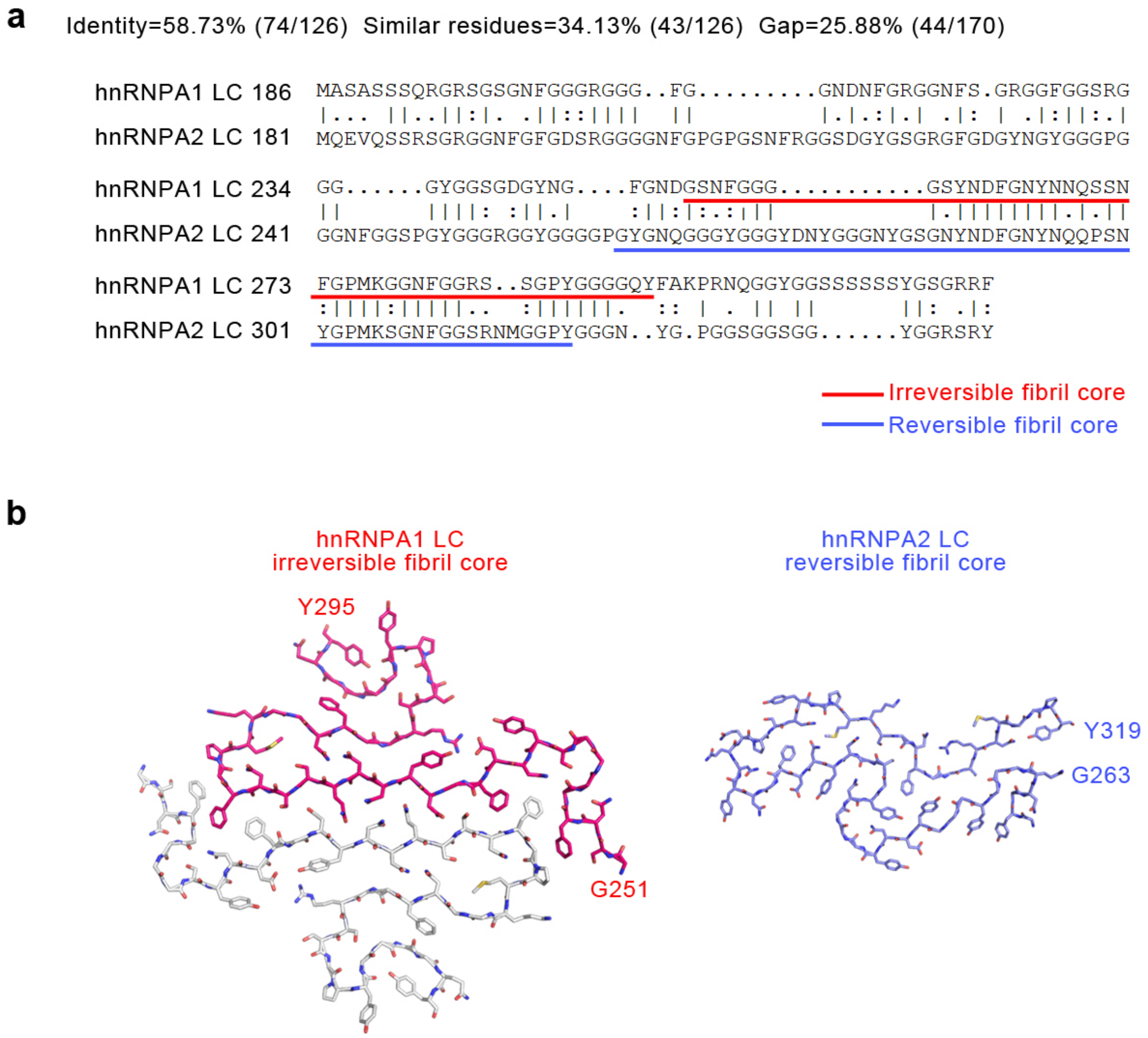

Supplementary Figure 9 Comparison of the reversible fibril of hnRNPA2 LC and the irreversible fibril of hnRNPA1 LC. a, Alignment of the primary sequence of the LC domain of hnRNPA1 and hnRNPA2 by DNAMAN software (Lynnon Biosoft). The segments that form the fibril core are underscored (hnRNPA1 in red; hnRNPA2 in blue). b, Top view of one layer of the fibril structures. HnRNPA1 LC forms a dimer; hnRNPA2 LC represents a monomer.

Supplementary Table 1 Buried surface areas of different amyloid fibril structures.

Fibril	PDB ID	Buried surface area (Å^2)
hnRNPA1 LC	This work	1,814.0
α-Synuclein		
Polymorph 1a	6A6B	705.0
Polymorph 1b	6CU8	789.4
MSA type I	6XYO	1,461.4*
MSA type II	6XYP	1,608.4*
Tau		
CBD type II	6TJX	324.6
CTE type I	6NWP	638.0
CTE type II	6NWQ	849.4

*There are co-factors bound in the interface between protofilaments.

Supplementary Table 2 Statistics of the Gibbs free energy changes upon mutations on hnRNPA1 LC fibrils.

Mutation	ΔΔG using ddg_monomer	ΔΔG using Cartesian_ddG
D262N	-17.482	-14.699
D262V	-32.924	-31.99
N267S	2.97	10.05
P288A	33.719	35.04
P288S	49.407	52.37

Supplementary Table 3 List of the primers used in this work.

Primer Name	Sequence (5’-3’)
F_ pET32M_Trx1_hnRNPA1 LCD_ D262N	CAATAACTTTGGGAATTACAACAATC
R_pET32M_Trx1_hnRNPA1 LCD_ D262N	CAAAGTTATTGTAGCTTCCACCAC
F_pET32M_Trx1_hnRNPA1 LCD_ D262V	CAATGTGTTTGGGAATTACAACAATC
R_pET32M_Trx1_hnRNPA1 LCD_ D262V	CAAACACATTGTAGCTTCCACCAC
F_pET32M_Trx1_hnRNPA1 LCD_ N267S	AATTACAGCAATCAGTCTTC
R_pET32M_Trx1_hnRNPA1 LCD_ N267S	TTGCTGTAATTCCCAAAATC
F_pET32M_Trx1_hnRNPA1 LCD_ P288A	GCAGAAGCTCTGGCGCGTATG
R_pET32M_Trx1_hnRNPA1 LCD_ P288A	CTCCACCGCCATACGCGC
F_pET32M_Trx1_hnRNPA1 LCD_ P288S	GCAGAAGCTCTGGCAGCTATG
R_pET32M_Trx1_hnRNPA1 LCD_ P288S	CTCCACCGCCATAGCTGC
