# Supplementary material for: The nuclear localization sequence mediates hnRNPA1 amyloid fibril formation revealed by cryoEM structure
Source: Nat Commun. 2020 Dec 11;11:6349. doi: 10.1038/s41467-020-20227-8 (PMC7733464; doi:10.1038/s41467-020-20227-8)
Supplement: Supplementary file 2 — Reporting Summary [file 41467_2020_20227_MOESM2_ESM.pdf]

nature research

Corresponding author(s): Cong Liu & Dan Li

Last updated by author(s): Nov 11, 2020

Reporting Summary

Nature Research wishes to improve the reproducibility of the work that we publish. This form provides structure for consistency and transparency in reporting. For further information on Nature Research policies, see our Editorial Policies and the Editorial Policy Checklist.

Statistics

For all statistical analyses, confirm that the following items are present in the figure legend, table legend, main text, or Methods section.

n/a	Confirmed	
☐	☒	The exact sample size (n) for each experimental group/condition, given as a discrete number and unit of measurement
☐	☒	A statement on whether measurements were taken from distinct samples or whether the same sample was measured repeatedly
☐	☒	The statistical test(s) used AND whether they are one- or two-sided<br>*Only common tests should be described solely by name; describe more complex techniques in the Methods section.*
☒	☐	A description of all covariates tested
☒	☐	A description of any assumptions or corrections, such as tests of normality and adjustment for multiple comparisons
☐	☒	A full description of the statistical parameters including central tendency (e.g. means) or other basic estimates (e.g. regression coefficient) AND variation (e.g. standard deviation) or associated estimates of uncertainty (e.g. confidence intervals)
☐	☒	For null hypothesis testing, the test statistic (e.g. F, t, r) with confidence intervals, effect sizes, degrees of freedom and P value noted<br>*Give P values as exact values whenever suitable.*
☒	☐	For Bayesian analysis, information on the choice of priors and Markov chain Monte Carlo settings
☒	☐	For hierarchical and complex designs, identification of the appropriate level for tests and full reporting of outcomes
☒	☐	Estimates of effect sizes (e.g. Cohen's d, Pearson's r), indicating how they were calculated

Our web collection on statistics for biologists contains articles on many of the points above.

Software and code

Policy information about availability of computer code

Data collection	SerialEM 3-7-3
Data analysis	NanoScope Analysis v1.5, ImageJ 2.0.0, GraphPad Prism 6, Relion v3.0, MotionCor2 v1.2.1, Ctffind v4.1.8, COOT v0.8.9.2, PHENIX v1.13, UCSF Chimera v1.13, Rosetta 2020.08, CCP4 package 7.0.076, Image Lab 3.0

For manuscripts utilizing custom algorithms or software that are central to the research but not yet described in published literature, software must be made available to editors and reviewers. We strongly encourage code deposition in a community repository (e.g. GitHub). See the Nature Research guidelines for submitting code & software for further information.

Data

Policy information about availability of data

All manuscripts must include a data availability statement. This statement should provide the following information, where applicable:

- Accession codes, unique identifiers, or web links for publicly available datasets
- A list of figures that have associated raw data
- A description of any restrictions on data availability

Density maps of the hnRNPA1 LC fibril are available through EMDB with entry code: EMD-30235. The structural model was deposited in the Protein Data Bank with entry code: 7BX7. Other structural models used in this study are available in the Protein Data Bank with entry codes: 2H4M (X-ray diffraction structure of Kapβ2), 1YJP (X-ray diffraction structure of GNNQQNY from yeast prion Sup35), 6RT0 (α-synuclein polymorph 1a), 6CU8 (α-synuclein polymorph 1b), 6XYO (α-synuclein MSA type I), 6XYP (α-synuclein MSA type II), 6TJX (Tau CBD type II), 6NWP (Tau CTE type I), and 6NWQ (Tau CTE type II). The source data underlying Supplementary Fig. 1b, 7b, 8b are provided as a Source Data file. Other data that support the findings of this study are available from the corresponding authors upon reasonable request.

Field-specific reporting

Please select the one below that is the best fit for your research. If you are not sure, read the appropriate sections before making your selection.

- [x] Life sciences
- [ ] Behavioural & social sciences
- [ ] Ecological, evolutionary & environmental sciences

For a reference copy of the document with all sections, see nature.com/documents/nr-reporting-summary-flat.pdf

Life sciences study design

All studies must disclose on these points even when the disclosure is negative.

Sample size	In the cytotoxicity experiment, 3 biologically independent replicates were applied for each sample. 3 independent experiments were performed, which provided reproducible results. As for structure determination, 3,227 cryoEM micrographs are of suitable quality for data processing. 62,656 fibrils were picked from these micrographs, which resulted in a nearly-atomic 3D density map.
Data exclusions	none
Replication	All experiments in this work including biochemical experiments, cytotoxicity experiment, and microscopic imaging, > 3 independent experiments were performed. All attempts at replication were successful.
Randomization	Randomization is not applicable for the experiments in this study. Indicated concentrations of proteins were used for biochemical and cellular experiments.
Blinding	Blinding is not applicable for the experiments in this study. Indicated concentrations of proteins were used for biochemical and cellular experiments. No animal or human studies were involved.

Reporting for specific materials, systems and methods

We require information from authors about some types of materials, experimental systems and methods used in many studies. Here, indicate whether each material, system or method listed is relevant to your study. If you are not sure if a list item applies to your research, read the appropriate section before selecting a response.

Materials & experimental systems

n/a	Involved in the study	
x		Antibodies
	x	Eukaryotic cell lines
x		Palaeontology and archaeology
x		Animals and other organisms
x		Human research participants
x		Clinical data
x		Dual use research of concern

Methods

n/a	Involved in the study	
x		ChIP-seq
x		Flow cytometry
x		MRI-based neuroimaging

Eukaryotic cell lines

Policy information about cell lines

Cell line source(s)	HEK-293T (Cat.# CRL-3216) cell lines were purchased from ATCC, USA.
Authentication	HKE-293T have been authenticated by STR method.
Mycoplasma contamination	The cell line is Mycoplasma negative.
Commonly misidentified lines (See ICLAC register)	No commonly misidentified cell lines were used.
